# The impact of social media on university students’ revisit intention in sports tourism: A hybrid method based on SEM and ANN

**DOI:** 10.1371/journal.pone.0321999

**Published:** 2025-04-29

**Authors:** Jinrong Li, Fang Li, Xiaomin Zhou

**Affiliations:** School of Physical Education, Hubei Minzu University, Enshi, Hubei, China; Sri Sivasubramaniya Nadar College of Engineering, INDIA

## Abstract

The primary aim of this study is to explore the influence of social media on university students’ revisit intention in sports tourism, using Expectation-Confirmation Model and the Uses and Gratifications Theory. A structured questionnaire was distributed to a random sample of 435 students from three universities in Hubei Province to measure their self-reported responses across six constructs: perceived usefulness, information quality, perceived enjoyment, electronic word-of-mouth (eWOM), satisfaction, and revisit intention. Employing a hybrid approach of Structural Equation Modeling (SEM) and Artificial Neural Networks (ANN), the study explains the non-compensatory and non-linear relationships between predictive factors and university students’ revisit intention in sports tourism. The results indicate that information quality, perceived enjoyment, satisfaction, and eWOM are significant direct predictors of revisit intention in sports tourism. In contrast, the direct influence of perceived usefulness on revisit intention is insignificant. ANN analysis revealed the normalized importance ranking of the predictors as follows: eWOM, information quality, satisfaction, perceived enjoyment, and perceived usefulness. This study not only provides new insights into the existing literature on the impact of social media on students’ tourism behavior but also serves as a valuable reference for future research on tourism behavior.

## 1. Introduction

In recent years, sports tourism, as a unique form of tourism, has garnered increasing attention. According to data from the United Nations World Tourism Organization (UNWTO), the global sports tourism market is projected to reach $50 billion by 2024 [1]. Revisit intention (RI), an essential indicator of tourist loyalty and the long-term attractiveness of a destination remains a relatively underexplored research area. RI refers to a tourist’s willingness to return to the same destination after their initial visit. Enhancing tourists’ RI is crucial for the sustainable development, reputation, and revenue growth of tourism destinations.

Existing studies have found that virtual reality technology [2], augmented reality technology [3], mobile technology [4], and artificial intelligence technology [5] have a significant impact on individuals’ travel decision-making behavior. These technologies reshape tourists’ decision-making process by altering how they acquire information, interact, and share experiences. Meanwhile, previous research has applied various theoretical frameworks, including the attitude-behavior theory [6], the motivation theory [7], the theory of planned behavior [8], and the sports value framework [9], to explain the formation mechanisms of individuals’ RI in sports tourism. Findings indicate that factors such as destination image, perceived quality, perceived value, and tourist satisfaction are significant predictors of RI in sports tourism [10,11]. However, despite the widespread adoption of social media, which has profoundly transformed tourists’ decision-making processes—particularly in terms of information acquisition, interaction, and experience sharing—research on how social media influences RI in sports tourism remains limited, especially concerning university students [12,13]. This research gap restricts a comprehensive understanding of social media’s potential in enhancing RI in sports tourism and poses challenges for destination managers in formulating effective strategies to leverage social media to increase tourist retention rates.

Drawing on the Uses and Gratifications Theory (U&GT) and the Expectation-Confirmation Model (ECM), this study employs Structural Equation Modeling (SEM) and Artificial Neural Network (ANN) analysis to examine and predict the impact of social media on university students’ RI in sports tourism. The selection of these two theoretical frameworks allows for an explanation of students’ behavior on social media from both satisfaction and motivational perspectives. The ECM helps elucidate how users’ satisfaction influences their RI in sports tourism by examining the gap between their initial expectations and actual experiences. Meanwhile, the U&GT explores how users’ motivations—such as entertainment, information acquisition, and social needs—drive their engagement with social media and, in turn, influence their RI. Specifically, this study operationalizes social media-related information acquisition through information quality, entertainment through perceived enjoyment, and social needs through electronic word-of-mouth (eWOM). By integrating these two models, this study provides a comprehensive understanding of the role of social media in shaping university students’ RI in sports tourism, taking into account both functional effectiveness and users’ subjective needs. Based on the research objectives, the following research questions are proposed:

(1) Which social media factors significantly influence university students’ RI in sports tourism?
(2) To what extent do social media factors explain university students’ RI in sports tourism?
(3) What is the standardized importance of each factor in predicting university students’ RI in sports tourism?

This study offers three key contributions. First, it integrates the ECM and the U&G within its theoretical framework. By combining these two models, the study provides a multi-dimensional analytical approach to understanding the role of social media in tourism behavior, offering a more comprehensive perspective on how users’ needs drive their travel decisions. Second, the study employs a hybrid analytical approach by integrating SEM and ANN techniques. While SEM effectively handles multivariate linear relationships and uncovers causal links, it has limitations in capturing complex non-linear interactions. ANN compensates for this drawback by detecting non-linear relationships and improving predictive accuracy. The combination of SEM and ANN deepens the analysis and enhances the precision of predictions, offering a robust methodological framework for studying complex behavioral patterns. Finally, this study focuses on university students and examines the critical role of social media in shaping their RI in sports tourism. As digital natives, university students exhibit unique social media usage patterns. Understanding how they utilize social media for information acquisition, entertainment, and eWOM in their decision-making process has significant practical implications for tourism marketing and destination management.

## 2. Literature review and hypothesis development

### 2.1. Uses and gratifications theory

U&GT, proposed by Trowbridge, aims to explain how users actively select media to fulfill specific needs[14]. This theory posits that users engage with media to satisfy their information, entertainment, and social needs [15]. Information needs refer to users’ desire to acquire knowledge, news, and factual content through media. Entertainment needs pertain to the enjoyment and relaxation users seek from media consumption. Social needs involve users’ engagement with media to interact with others and establish social connections [15]. In recent years, with the rapid advancement of media technologies, U&GT has been widely applied across various modern media contexts, including social media [16], short video platforms [17], mobile technology [18], and virtual goods purchases [19].

Regarding information needs, research indicates that users’ demand for acquiring knowledge and news through media has become increasingly prominent in information explosion [15]. For example, Wu found that users of short video platforms fulfill their information needs by consuming educational content, enhancing their cross-cultural awareness [17]. However, existing studies primarily focus on general user groups, with limited research on the information needs of specific populations, such as university students. Moreover, most studies rely on self-reported data, which may introduce social desirability bias. Future research could employ behavioral data analysis and other methodologies to enhance the reliability of findings.

Regarding entertainment needs, research on short video platforms and virtual goods purchasing suggests that users seek pleasure and relaxation through media consumption [17]. However, the fulfillment of entertainment needs may vary based on cultural backgrounds and user demographics. For example, Wu found that while Chinese audiences satisfy their entertainment needs through short video platforms, they also enhance their cross-cultural awareness [17]. In contrast, Kaur et al. demonstrated that virtual goods purchasing is more closely linked to identity formation [19].

Regarding social needs, Sheldon found that older adults rely on social media to compensate for the lack of social interactions in real life [16]. However, research on the social needs of younger populations, such as university students, remains limited and is primarily focused on traditional social media platforms, with insufficient attention to emerging platforms like short video platforms. Additionally, most existing studies adopt a cross-sectional design, making capturing the dynamic changes in social needs over time difficult.

The U&GT is often integrated with other theoretical models to provide a more comprehensive explanation of users’ complex motivations and needs for media consumption. For example, Florenthal combined it with the Technology Acceptance Model to uncover the motivations behind users’ technology adoption behavior [20]. Guo et al. integrated it with the Stimulus-Organism-Response model to analyze users’ emotional responses to media content [21]. Ledbetter et al applied it alongside the Media Multiplexity Theory to explore behavioral differences across various media platforms [22]. Similarly, Zadeh et al. incorporated it into the Theory of Planned Behavior to reveal the relationship between users’ attitudes and behavioral intentions [23]. Although the U&GT has made significant progress in explaining users’ media usage motivations, research focusing on specific user groups, such as university students, and emerging media platforms, such as social media, remains limited. This gap constrains the generalizability of the theory.

### 2.2. Expectation confirmation model

**Fig 1:**
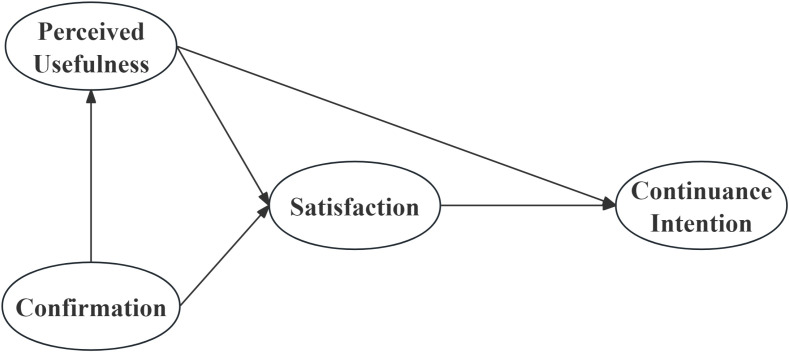
Expectation confirmation model.

The ECM, proposed by Bhattacherjee, is primarily used to examine user satisfaction with information systems and their continuance intention [24]. The model comprises four key constructs: perceived usefulness, confirmation, satisfaction, and continuance intention (Fig 1). According to Bhattacherjee, perceived usefulness refers to users’ subjective evaluation of the value and significance of an information system in practical application; confirmation represents the extent to which users perceive consistency between their initial expectations and the system’s actual performance; satisfaction reflects users’ overall subjective experience and evaluation of the system after use; and continuance intention denotes their inclination to continue using the system [24]. Based on ECM, perceived usefulness, confirmation, and satisfaction are critical factors influencing users’ continuance intention [25,26].

In recent years, the ECM has been validated and extended across various domains, including health education videos, AI-powered drawing applications [27], fitness wearables [28], online sports videos [29], and virtual experience platforms [30]. For instance, based on the cognitive-affective-intentional framework, Xuan et al. found that user satisfaction and trust in health education videos significantly and positively influence continuance intention [31]. Yu et al. incorporated flow experience and social influence into ECM and found that social influence was the strongest predictor of continuance intention [27]. However, the fundamental ECM framework has certain limitations. For example, measuring perceived usefulness and satisfaction varies considerably across studies, reducing the comparability of findings [32]. Additionally, most studies focus on East Asian markets, with limited cross-cultural validation [30]. To enhance ECM’s predictive power, scholars have expanded the model by introducing new variables (e.g., perceived value, technological stress, habit) or integrating it with other theoretical frameworks (e.g., TAM, UTAUT2). For example, Kim et al. identified performance expectancy and hedonic motivation as key predictors in virtual experience platform research [30], while Yu et al. demonstrated that habit moderates the relationship between satisfaction and continuance intention, strengthening the model’s explanatory power in AI-powered drawing applications [27]. Despite these advancements, existing ECM extensions primarily focus on single domains, lacking an exploration of cross-domain commonalities. Moreover, the integration pathways between ECM and other theories require further validation, particularly in emerging technological fields such as generative AI. Addressing these research gaps, the present study applies ECM to the sports tourism context among university students, examining the mechanisms through which social media influences RI. The findings aim to provide actionable ECM-based user retention strategies for sports tourism practitioners, such as enhancing user satisfaction to increase engagement and loyalty. By systematically reviewing ECM’s foundational research, extended applications, and theoretical integrations, this study highlights both its potential and limitations in explaining continuance intention and offers new directions for future research.

### 2.3. Revisit intention

RI, a key indicator for predicting tourists’ future return decisions, is an individual’s willingness to revisit and recommend the same destination [33]. Research on RI has expanded beyond traditional tourism destinations to fields such as medical tourism [34] and hotel services, drawing on various theoretical frameworks, including the Theory of Planned Behavior, the Stimulus-Organism-Response model, and Flow Theory. Studies indicate that RI is influenced by internal factors (e.g., destination image, tourist satisfaction) and external factors (e.g., digital technology applications, social environment). For instance, Rahman et al. found that the cultural attractiveness of a destination enhances RI through emotional attachment [34], while Ouyang et al. demonstrated that innovative tourism technologies indirectly drive revisit behavior by enhancing tourists’ perceived experiences [35]. However, existing research has notable limitations. First, most studies adopt a single-theory perspective, lacking an integrative analysis of the interactive effects among multiple influencing factors [36]. Second, the research context remains heavily focused on traditional tourism, with limited attention to emerging domains such as sports tourism. Addressing these gaps, the present study extends RI research to the university student sports tourism context by integrating the U&GT and the ECM. This study explores the interactive influence of social media on RI, aiming to provide precise user retention strategies for industry practitioners by enhancing tourist satisfaction. Furthermore, this research contributes to the theoretical development of RI by expanding its application into multidisciplinary and technology-driven contexts.

### 2.4. Perceived usefulness, satisfaction, and RI

Numerous studies have confirmed the significant influence of perceived usefulness, satisfaction, and continuance intention [27,31,37–40]. For example, L. Pan et al. investigated the impact of online sports videos on tourists’ RI [38]. They found that perceived usefulness and satisfaction significantly influenced tourists’ intention to revisit rural destinations. Similarly, Tian et al. examined factors influencing Chinese postgraduate students’ intention to use smart chatbot technology and found that user satisfaction directly and positively reinforced their intention [39]. Furthermore, Yu et al. studied the factors influencing the continuance intention of Korean learners in MOOCs [27]. They concluded that perceived usefulness and satisfaction significantly and positively impacted their intention to use MOOCs. Consequently, this study hypothesizes that when university students use social media tools during sports tourism and perceive that these tools enhance their travel efficiency and experience, their perceived usefulness increases, directly improving their satisfaction and strengthening their RI. Therefore, the following hypotheses are proposed:

**H1:** University students’ perceived usefulness of social media significantly and positively influences their RI in sports tourism.
**H2:** University students’ perceived usefulness of social media significantly and positively influences their satisfaction in sports tourism.
**H3:** University students’ satisfaction with social media significantly and positively influences their RI in sports tourism.

### 2.5. Information quality, satisfaction, and RI

Information quality refers to the characteristics of the system’s output, which should be accurate, complete, and timely [41]. Numerous studies have confirmed that information quality predicts user satisfaction and RI [28,31,32,42]. For example, based on the Cognitive-Affective-Conative Model and the ECM, Xuan et al. analyzed the impact of health education videos on user satisfaction and continuance intention, finding that video information quality was a significant predictor [31]. Similarly, Lee et al. investigated the factors influencing Korean learners’ continuance intention to use MOOC platforms, and their results showed that the quality of information provided by MOOCs significantly predicted user satisfaction, thereby enhancing continuance intention [32]. According to previous findings, this study hypothesizes that in university students’ sports tourism, the higher the quality of information provided by social media, the greater their satisfaction with the media and the stronger their RI toward the tourism destination. Therefore, the following hypotheses are proposed:

**H4:** Information quality provided by social media significantly and positively influences university students’ satisfaction in sports tourism.
**H5:** Information quality provided by social media significantly and positively influences university students’ RI in sports tourism.

### 2.6. Perceived enjoyment, satisfaction, and RI

Perceived enjoyment refers to the pleasure and excitement derived from using social media [43]. Perceived enjoyment has been shown to significantly impact both satisfaction and RI [28,44,45]. Kim et al. examined the relationship between perceived usefulness, perceived enjoyment, perceived responsiveness, and continuance intention of ChatGPT [46]. Their findings revealed that both perceived usefulness and enjoyment significantly and positively influenced satisfaction with ChatGPT, significantly and positively affecting users’ continuance intention [46]. Zhan et al. studied the relationship between urban park attractiveness, visitor perceptions, and RI and found that the attractiveness of urban parks significantly and positively influenced visitor satisfaction [45]. On the other hand, Sun and Gu investigated the determinants of continued use of wearable fitness devices [28]. They found that perceived enjoyment (such as technological innovation and device functionality) impacted consumers’ intention to continue using wearable fitness devices. According to previous findings, this study hypothesizes that during sports tourism, the stronger university students’ perceived enjoyment of social media, the greater their perceived usefulness and satisfaction, and the stronger their RI toward the tourism destination. Therefore, the following hypotheses are proposed:

**H6:** University students’ perceived enjoyment of social media significantly and positively influences satisfaction in sports tourism.
**H7:** University students’ perceived enjoyment of social media significantly and positively influences RI in sports tourism.

### 2.7. eWOM, satisfaction, and RI

eWOM refers to users sharing information on social media platforms, which spreads through social networks and influences others’ perceptions and decisions [47]. eWOM is also an essential factor influencing user satisfaction and continuance intention [48–50]. For instance, Salah et al. examined the impact of eWOM on RI in five-star hotels in Saudi Arabia, revealing that eWOM had a significant positive impact on RI [49]. Kanwel et al. explored the effect of destination image and tourist loyalty in Pakistan, proposing that eWOM could enhance tourist satisfaction and influence their RI [48]. Similarly, Yoo studied the factors affecting consumers’ eWOM revision behaviors and repurchase intention, suggesting that eWOM significantly and positively influences both satisfaction and repurchase intention [50]. Based on these studies, this research hypothesizes that during sports tourism, the better the eWOM on social media, the stronger the users’ satisfaction and the greater their RI toward the destination. Therefore, the following hypotheses are proposed:

**H8:** The eWOM on social media significantly and positively influences university students’ satisfaction in sports tourism.
**H9:** The eWOM on social media significantly and positively influences university students’ RI in sports tourism.

**Fig 2:**
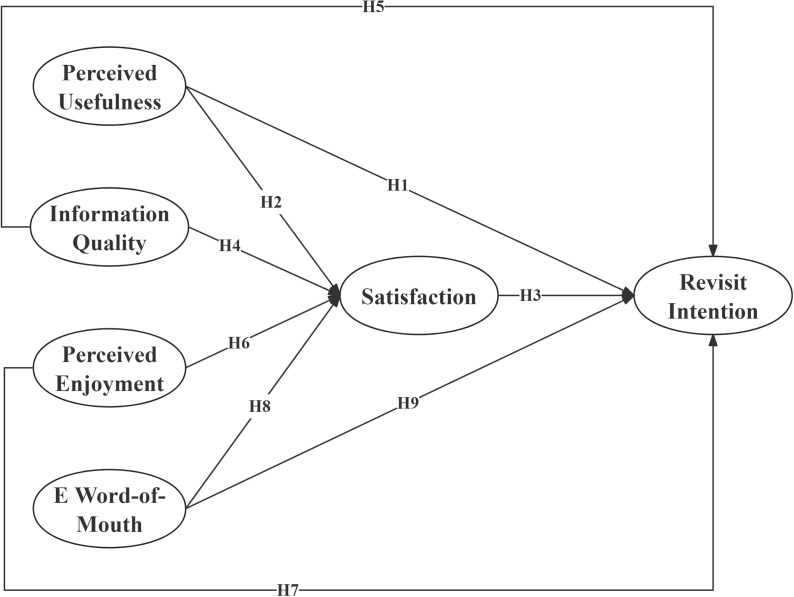
Hypothesized model.

Based on the above hypotheses, the hypothesized model of this study is proposed (Fig 2).

## 3. Methodology

### 3.1. Sample and data collection

This study collected data from April to July 2024 using the Questionnaire Star online survey platform (www.Sojump.com) to test the proposed hypothesis model. Stratified random sampling was employed to improve the representativeness and precision of the sample, particularly in studies requiring high accuracy and handling complex data [51]. This method has demonstrated significant advantages in reducing variance and enhancing statistical power [52]. The researchers confirm that all research was performed in accordance with relevant guidelines/regulations applicable when human participants are involved (e.g., Declaration of Helsinki or similar). This study was approved by the Ethics Committee of Hubei Minzu University, with the approval number HMU-2024-03-0006.

This study adopted a stratified random sampling approach to ensure a representative sample, selecting participants from three universities in Hubei Province: Hubei Minzu University, Hubei Enshi College, and Enshi Vocational and Technical College. First, students were stratified by academic year. Within each stratum, students were randomly selected to participate in the survey, ensuring equal opportunity for selection and thereby enhancing the representativeness and reliability of the sample. Next, within each academic year, researchers used a random number generator to select 150 students from the enrollment list randomly and emailed them survey invitations. Before participating, students were required to read an informed consent form outlining the research objectives, data usage, and their rights. After confirming their consent, they completed and submitted the questionnaire.

By the end of the data collection, 472 responses were gathered. To ensure the representativeness and validity of the sample, the following inclusion criteria were applied: First, participants had to be full-time university students across all academic years to ensure diversity and representativeness. Second, students from Hubei Province were selected to control for potential geographical and cultural influences on the research findings. Third, all participants voluntarily participated and clearly understood the study’s objectives and requirements. Finally, all participants signed an informed consent form before completing the survey, ensuring compliance with ethical and legal research standards.

After data screening, 435 valid responses remained for analysis. In the research model of this study, the maximum number of arrows pointing to an endogenous latent construct was five. According to Hair [53], to achieve an R^2^ value greater than 0.10 with a 1% significance level, the minimum required sample size is 205. The use of 435 valid responses significantly exceeds this threshold, ensuring the robustness and reliability of the findings.

The sample comprised 187 male students (42.99%) and 248 female students (57.01%) (Table 1). Regarding academic background, 130 participants (29.89%) were sports-related majors, while 305 (70.11%) were non-sports majors. The distribution of students by academic year was as follows: 154 freshmen (35.40%); 159 sophomore (36.55%); 74 junior (17.01%); 48 senior (11.03%). This representative sample provides a solid foundation for analyzing the relationships between university students’ perceptions of social media’s perceived usefulness, information quality, perceived enjoyment, eWOM, satisfaction, and RI in sports tourism. The sample diversity and size enhance the generalizability of the study’s findings within this research context, allowing for broader applicability to the university student population.

**Table 1:** Demographic characteristics of the sample.

Demographic	Category	Frequency	Percentage
Gender	Male	187	42.99%
	Female	248	57.01%
Grade	Freshmen	154	35.40%
	Sophomore	159	36.55%
	Junior	74	17.01%
	Senior	48	11.03%
Major	Sports major	130	29.89%
	Non-sports major	305	70.11%

### 3.2. Instruments

The questionnaire was divided into two main sections: the first section collected demographic information from participants, and the second section gathered self-reported data on various constructs. Validated scales were used to assess these constructs, adjusting to fit the research context and objectives. For example, in the satisfaction construct, the original item “I am very satisfied after using this learning system” was modified to “I am satisfied with my sports travel experience after using social media.” This adjustment ensures that the questions better reflect participants’ experiences within the study’s specific context. All original English questionnaires were translated into Chinese following the translation and back-translation procedure proposed by Beaton et al. [54]. Precisely, this study’s first and second authors, proficient in English, translated the scales into Chinese. Subsequently, the third author back-translated all scales into English to verify translation accuracy. Additionally, a bilingual faculty member from a U.S. university reviewed the translated and back-translated versions of the scales to ensure their accuracy further. The final survey comprised demographic information (e.g., gender, age) and all measurement scales for the study’s constructs.

In addition to basic demographic information, the questionnaire included six key constructs: perceived usefulness, information quality, perceived enjoyment, eWOM, satisfaction, and RI. Compared to a seven-point Likert scale, a five-point Likert scale has significant advantages in enhancing the reliability and validity of the scale [55], reducing bias, and improving the sensitivity of statistical analyses [56]. Therefore, each construct was measured using a five-point Likert scale, ranging from (1) “strongly disagree” to (5) “strongly agree.”

Perceived usefulness was measured using a scale developed by Al-Adwan et al. [57]. In this study, the scale was used to assess university students’ perceptions of the usefulness of social media. The scale consisted of four items (e.g., “I believe that social media enhances the quality of sports travel”). The Cronbach’s α for this scale was 0.864, indicating good reliability.

Information quality was measured using a scale by Idkhan and Idris [58]. This study used the scale to assess university students’ perceptions of the quality of information provided by social media. The scale consisted of three items (e.g., “I believe that social media presents information clearly and understandably”). The Cronbach’s α for this scale was 0.863, indicating good reliability.

Perceived enjoyment was measured using a scale developed by Al-Adwan et al. [57]. In this study, the scale was used to assess university students’ perceptions of the entertainment value of social media. The scale consisted of four items (e.g., “Using social media makes sports travel more enjoyable”). The Cronbach’s α for this scale was 0.900, indicating excellent reliability.

eWOM was measured using a scale developed by Xu et al. [59]. This study used the scale to assess university students’ perceptions of the influence of social media reviews. The scale consisted of six items (e.g., “Through social media, eWOM can encourage university students’ sports travel behavior”). The Cronbach’s α for this scale was 0.942, indicating excellent reliability.

Satisfaction was measured using a scale developed by Idkhan and Idris [58]. This study used the scale to assess university students’ perceptions of whether social media meets their needs. The scale consisted of four items (e.g., “I am satisfied with the overall functionality of social media”). The Cronbach’s α for this scale was 0.898, indicating good reliability.

RI was measured using a scale developed by Nazarian et al. [60]. This study used the scale to assess the strength of university students’ RI in sports tourism. The scale consisted of three items (e.g., “In the future, I will continue using social media for sports tourism”). The Cronbach’s α for this scale was 0.875, indicating good reliability. For detailed measurement items, refer to Appendix A in S1 File.

### 3.3. Data analysis

This study employed a two-stage approach to test the hypotheses. First, Partial Least Squares Structural Equation Modeling (PLS-SEM) was used to identify the linear relationships between exogenous and endogenous variables. PLS-SEM has strong advantages in handling small sample sizes, non-normal data, exploratory research, and complex models[53]. With a sample size of only 435 and including 6 constructs and 24 items, this study falls under exploratory research involving a complex model [61]. Therefore, PLS-SEM is well-suited for the data analysis in this study.

However, SEM has limitations in capturing non-linear and non-compensatory relationships, which are crucial for understanding the complex dynamics of how social media influences university students’ RI in sports tourism.

To address these limitations, the second stage of this study integrated ANN. ANN can capture linear and non-linear relationships using non-compensatory models, thus improving predictive accuracy [62,63]. These studies highlight the robustness of ANN in handling complex data patterns and predictive tasks. By combining SEM and ANN, this study enhances the depth and accuracy of data analysis, providing a comprehensive understanding of how social media influences university students’ RI in sports tourism.

## 4. Results

Researchers employed various statistical methods to develop and test the research hypotheses. Hair distinguish between the applications of first-generation and second-generation statistical methods [64]. Factor analysis and regression analysis dominated first-generation methods and were widely used. Since the 1990s, more advanced multivariate statistical methods, such as SEM, have become dominant in second-generation methods (Goggins and Xing, 2016). SEM has two main types: covariance-Based SEM and Variance-Based SEM (PLS-SEM). Given the complexity of the model in this study—6 constructs, 24 items, and nine relationships—PLS-SEM is well-suited for analyzing such complex models [64].In this study, SmartPLS 4.0 was used to evaluate both the measurement model and the structural model.

### 4.1. Measurement model

The evaluation of the measurement model followed these criteria:

- Step 1: Indicator Reliability: The outer loadings of the indicators should be ≥ 0.70 [64].
- Step 2: Internal Consistency Reliability: Two methods were used to assess this—Cronbach’s alpha (α) and Composite Reliability (CR). The threshold for both tests is ≥ 0.70 [65].
- Step 3: Validity:
  Convergent Validity: The Average Variance Extracted (AVE) should be ≥ 0.50 [66].
  Discriminant Validity: Two methods were used to assess this:

- First, the Fornell-Larcker criterion [66].
- Second, the Heterotrait-Monotrait Ratio (HTMT) [67].

First, internal consistency reliability and validity were evaluated through Cronbach’s alpha, CR, and AVE. As shown in Table 2, all values met the minimum thresholds for indicator reliability and internal consistency reliability. Furthermore, the AVE for all constructs was ≥ 0.50, confirming the convergent validity of the model.

**Table 2:** Reliability and AVE.

Constructs	Items	Outer loadings	Cronbach’s α	CR	AVE
IQ	IQ1	0.866	0.863	0.954	0.777
	IQ2	0.896			
	IQ3	0.895			
PE	PE1	0.879	0.900	0.916	0.785
	PE2	0.898			
	PE3	0.908			
	PE4	0.823			
PU	PU1	0.79	0.864	0.931	0.770
	PU2	0.821			
	PU3	0.868			
	PU4	0.888			
RI	RI1	0.861	0.875	0.907	0.71
	RI2	0.914			
	RI3	0.907			
SAT	SAT1	0.862	0.898	0.923	0.800
	SAT2	0.872			
	SAT3	0.875			
	SAT4	0.893			
eWoM	eWoM1	0.898	0.942	0.929	0.766
	eWoM2	0.904			
	eWoM3	0.901			
	eWoM4	0.885			
	eWoM5	0.849			
	eWoM6	0.85			

Next, the correlation matrix for the Fornell-Larcker discriminant validity test is presented in Table 3. According to Hair [53], the square root of each construct’s AVE should be higher than the highest correlation between that construct and any other construct in the model. As shown in Table 3, the results meet this criterion, confirming the discriminant validity of the constructs in the model.

**Table 3:** Discriminant validity (Fornell-Larcker criteria).

	eWoM	IQ	PE	PU	RI	SAT
eWoM	**0.881**					
IQ	0.679	**0.886**				
PE	0.738	0.713	**0.878**			
PU	0.714	0.657	0.779	**0.843**		
RI	0.804	0.716	0.723	0.698	**0.895**	
SAT	0.838	0.779	0.745	0.74	0.779	**0.875**

Note: The bolded values on the diagonal represent the square roots of the AVE for each construct.

The Heterotrait-Monotrait Ratio (HTMT), proposed by Henseler et al. [67], is a criterion used to assess discriminant validity. HTMT is calculated as the average of the heterotrait-heteromethod correlations relative to the monotrait-heteromethod correlations. Heterotrait-heteromethod correlations measure the relationships between indicators across different constructs, while monotrait-heteromethod correlations measure the relationships between indicators of the same construct.

The HTMT values were calculated using SmartPLS software and are presented in Table 4. All HTMT values fall within the acceptable threshold of ≤0.90, as recommended by Henseler et al. [67], confirming the discriminant validity of the constructs.

**Table 4:** Discriminant validity (HTMT criteria).

	eWoM	IQ	PE	PU	RI	SAT
eWoM						
IQ	0.751					
PE	0.802	0.809				
PU	0.788	0.755	0.881			
RI	0.883	0.823	0.814	0.799		
SAT	0.816	0.883	0.828	0.834	0.877	

### 4.2. Structural model

The evaluation of the structural model followed the steps recommended by Hair [53], using the following criteria:

1. Assess collinearity issues (VIF < 5).
2. Assess the significance and relevance of structural model relationships (p < 0.05).
3. Assess the coefficient of determination (R^2^) level with thresholds: 0.190 for weak, 0.333 for moderate, and 0.670 for strong.
4. Assess the predictive relevance (Q^2^) level with a threshold greater than zero.

First, collinearity was assessed using the Variance Inflation Factor (VIF). A VIF value of ≥5 indicates potential collinearity issues. In this study, all VIF values were within the acceptable threshold (VIF < 5), as shown in Table 5. Therefore, no collinearity issues were present in the data.

**Table 5:** VIF.

	eWoM	IQ	PE	PU	RI	SAT
eWoM					3.888	2.656
IQ					2.84	2.318
PE					3.413	3.407
PU					3.021	2.909
RI						
SAT					4.075	

Secondly, the path coefficients (β) between the constructs in the model are shown in Table 6. The significance of the path coefficients is assessed using the bootstrapping algorithm in PLS. The t-values and p-values are used to test whether the path coefficient is statistically significant at the 5% level. A 5% significance level indicates that the p-value must be less than 0.05 and the t-value must be greater than 1.96. The results of the bootstrapping algorithm are presented in Table 6.

**Table 6:** Path analysis and hypothesis testing.

Hypothesis	β coefficients	Standard deviation	T statistics	P values	Results
IQ → RI	0.198	0.047	4.23	0.000	Supported
IQ → SAT	0.321	0.039	8.199	0.000	Supported
PE → RI	0.11	0.054	2.042	0.041	Supported
PE → SAT	0.037	0.069	0.536	0.592	Not Supported
PU → RI	0.094	0.055	1.696	0.090	Not Supported
PU → SAT	0.148	0.05	2.963	0.003	Supported
SAT → RI	0.116	0.066	4.247	0.000	Supported
eWoM → RI	0.423	0.058	7.299	0.000	Supported
eWoM → SAT	0.493	0.051	9.597	0.000	Supported

Third, the R^2^ represents the variance in the endogenous construct explained by all related exogenous constructs. Values of approximately 0.67 are considered substantial, around 0.33 are moderate, and around 0.19 are weak. Perceived usefulness, information quality, perceived enjoyment, and eWOM collectively explain 71.7% of the variance in RI. Additionally, perceived usefulness, information quality, perceived enjoyment, and eWOM explain 80.3% of the variance in satisfaction.

Fourth, the blindfolding method in SmartPLS was used to calculate the Q^2^ value, which evaluates the predictive relevance of the model. In a structural model, a Q^2^ value greater than zero indicates that the model has predictive relevance for a specific endogenous latent construct [53]. Table 7 shows the path model’s predictive relevance for the endogenous latent constructs.

**Table 7:** R^2^ and Q^2^.

Constructs	R^2^	Q^2^
SAT	80.3%	0.607
RI	71.7%	0.565

### 4.3. ANN analysis

This study used the significant factors identified in the SEM-PLS path analysis as input neurons in the ANN model. The rationale for applying ANN includes the data’s non-normal distribution and non-linear relationships between exogenous and endogenous variables. Additionally, ANN is robust against noise, outliers, and small sample sizes. It is also suitable for non-compensatory models, where a reduction in one factor does not need to be compensated by an increase in another. The ANN analysis was performed using IBM’s SPSS Neural Network module.

The ANN algorithm can capture both linear and non-linear relationships without requiring a normal distribution [68]. The algorithm learns through a training process using the feedforward-backpropagation (FFBP) algorithm to predict outcomes [69]. Multilayer perceptrons and sigmoid activation functions were used for the input and hidden layers [70]. Through multiple learning iterations, errors are minimized, improving prediction accuracy further [71]. In this study, 70% of the sample was used for training, while the remaining sample was used for testing.

To avoid the possibility of overfitting, a ten-fold cross-validation procedure was conducted, and the root mean square error (RMSE) was calculated [72]. As shown in Table 8, the average RMSE values for the training and testing processes were 0.0964 and 0.1005, respectively, indicating an excellent model fit.

**Table 8:** Root mean square of error values.

Training			Testing			Total samples
N	SSE	RMSE	N	SSE	RMSE	
296	2.8409	0.0980	139	1.2085	0.0932	435
315	2.6609	0.0919	120	1.6093	0.1158	435
294	2.7396	0.0965	141	1.3248	0.0969	435
292	2.8469	0.0987	143	1.367	0.0978	435
298	2.8923	0.0985	137	1.201	0.0936	435
314	3.0965	0.0993	121	1.0382	0.0926	435
297	2.6916	0.0952	138	1.4360	0.1020	435
304	2.4602	0.0900	131	1.540	0.1084	435
307	2.8962	0.0971	128	1.251	0.0989	435
317	3.0961	0.0988	118	1.322	0.1058	435
Mean	2.822116667	0.0964	Mean	1.3297	0.1005	
Sd		0.0032	Sd		0.0075	

Note: N: number of samples; SSE: sum square of error; RMSE: root mean square of error.

To assess the predictive strength of each input neuron, we conducted a sensitivity analysis (Table 9). The normalized importance of these neurons was obtained by dividing their relative importance by the highest importance and presenting it as a percentage [73]. The results show that eWOM is the most important predictor, with a normalized importance of 100%. This is followed by information quality with a normalized importance of 47.1%, satisfaction (42.5%), perceived enjoyment (39.6%), and perceived usefulness (31.3%).

**Table 9:** Sensitivity analysis.

ANN	eWoM	IQ	PE	PU	SAT
ANN 1	1.000	0.577	0.319	0.343	0.357
ANN 2	1.000	0.465	0.227	0.282	0.261
ANN 3	1.000	0.405	0.384	0.106	0.355
ANN 4	1.000	0.575	0.344	0.192	0.186
ANN 5	1.000	0.582	0.564	0.397	0.594
ANN 6	1.000	0.282	0.256	0.308	0.389
ANN 7	1.000	0.402	0.306	0.285	0.294
ANN 8	0.898	0.448	0.688	0.418	1.000
ANN 9	1.000	0.394	0.412	0.197	0.529
ANN 10	1.000	0.534	0.420	0.569	0.243
Mean importance	0.990	0.466	0.392	0.310	0.421
Normalized importance (%)	100.0%	47.1%	39.6%	31.3%	42.5%

## 5. Discussion

In sports tourism, the most significant direct predictor of university students’ RI is eWOM on social media, followed by information quality, satisfaction, and perceived enjoyment. First, eWOM on social media has a significant positive impact on RI, consistent with the findings of Salah et al. [49]. eWOM originates from real user experiences, and feedback is often more credible than official promotions. Consumers are more inclined to trust reviews and recommendations from other users, thus enhancing their trust in the destination, a critical factor in increasing RI. Second, the information quality on social media significantly influences RI in sports tourism, aligning with the findings of Torabi et al. [74]. High-quality social media information helps university students make better decisions, triggering cognitive and emotional resonance and strengthening their RI. Third, satisfaction with social media has a significant positive impact on RI, consistent with the research of Zhao and Liu, which found that satisfaction on the popular review site Dianping significantly influences restaurant RI [36]. University students’ satisfaction with social media positively affects their RI. The satisfaction gained from positive interactions and rich content experiences on social media enhances students’ emotional connection to the destination. It effectively improves the destination’s image, contributing to a higher likelihood of revisiting.

Finally, perceived enjoyment of social media during sports tourism has a significant positive impact on RI, consistent with the findings of Ibrahim et al. [75]. In this study, perceived enjoyment of social media positively affects university students’ RI in sports tourism. Entertaining social media content highlights the uniqueness and attractiveness of the destination, creating a more positive image in users’ minds, increasing their desire to visit, and ultimately enhancing their RI.

However, the perceived usefulness of social media does not directly affect RI in tourism, which is inconsistent with the findings of Torabi et al. [40] and Yu et al. [27]. This discrepancy may be attributed to the interactive effects of perceived usefulness, other factors, and the differences in the purpose of social media usage. First, perceived usefulness may indirectly influence RI through other mediating variables, such as attitude, trust, and satisfaction, rather than exerting a direct effect. This indirect influence may vary significantly across studies [76]. Second, the primary purpose for which university students use social media—whether for socializing, entertainment, or information gathering—could affect the degree to which they value the platform’s perceived usefulness. If the primary purpose of the use is entertainment or socializing, the suitability of the information might have a lesser impact on RI [77].

Among all the direct predictors of satisfaction, eWOM is the most influential factor, followed by information quality and perceived usefulness. First, eWOM on social media has a significant positive effect on satisfaction, consistent with the findings of Baghirov et al. [78]. When deciding on a destination, users often refer to other tourists’ reviews and experiences, which can increase students’ satisfaction with social media. Second, the quality of information on social media significantly and positively affects satisfaction, consistent with the findings of Baghirov et al. [78]. In this study, when users perceive the information as reliable, they are more likely to feel satisfied with and continue using the platform. Third, the perceived usefulness of social media has a significant positive effect on satisfaction, consistent with the findings of Kim et al. [46]. In this study, the vast amount of instant information provided by social media helps university students quickly access destination-related information, thereby increasing their satisfaction with the platform.

However, perceived enjoyment does not significantly enhance university students’ satisfaction with social media, which is inconsistent with the findings of Kim et al. [46] and Huang and Yu [44]. This discrepancy may stem from differences in sample characteristics, contextual factors, the type of social media platforms used, and variations in how entertainment and satisfaction are perceived. First, this study’s sample characteristics and context might differ from previous studies. For instance, university students from different regions may have varying social media usage habits, preferences, and expectations, which could influence how they perceive enjoyment on social media and how it affects their satisfaction [79]. Second, university students’ perceptions of social media’s entertainment value and impact on satisfaction may vary. Some students may prioritize the functionality and practicality of social media over its entertainment value, thereby weakening the influence of entertainment on their satisfaction [77].

In the sports tourism, to better explore the roles of perceived usefulness, information quality, perceived enjoyment, eWOM, and satisfaction in predicting university students’ RI, this study applied ANN analysis, revealing the importance of these factors. The ANN results confirmed the findings of the SEM analysis and supplemented them. The ANN analysis showed that eWOM, information quality, and satisfaction are the three most important factors influencing university students’ RI to a destination. In sports tourism, eWOM on social media is the strongest predictor of university students’ RI. This finding is supported by Cheung and Thadani [80], who demonstrated that eWOM can directly enhance customer RI and indirectly do so through satisfaction. Information quality on social media plays the second most important role in predicting university students’ RI. This is consistent with previous literature [32,81]. Lee et al. found that the quality of information on MOOC platforms directly increases Korean learners’ continued intention and indirectly enhances it through satisfaction [32]. Satisfaction with social media ranks as the third most important factor in predicting university students’ RI. This viewpoint is supported by prior research [40,82]. indicated that the higher Iranian users’ satisfaction with innovative tourism technology, the higher their RI to emerging rural tourism destinations.

### 5.1. Implications

#### 5.1.1. Theoretical implications.

This study makes several significant theoretical contributions to sports tourism and social media research by addressing critical gaps in the literature and advancing our understanding of the mechanisms through which social media influences university students’ RI.

***First, advancing the understanding of the role of social media in sports tourism*:** This study provides a novel perspective by exploring how social media influences university students’ RI in the sports tourism. While previous research has examined the overall impact of social media on tourism behavior, the specific mechanisms through which social media affects university students—a demographic of digital natives—remain underexplored. The findings of this study reveal that university students’ unique social media usage patterns, including their reliance on platform information, entertainment, and social interaction, significantly shape their decision-making process and RI. This study fills a critical gap in the literature by linking this demographic to RI in sports tourism. It expands the scope of research on the relationship between social media and tourism behavior.

***Second, integrating the ECM and the U&GT*:** A key theoretical contribution of this study lies in combining the ECM and the U&GT to explain the role of social media in shaping university students’ RI. U&GT provides a foundational framework for understanding users’ motivations for engaging with social media, emphasizing the fulfillment of informational, entertainment, and social needs. In contrast, ECM highlights the key factors driving the continued use of social media, such as confirmation, perceived usefulness, and satisfaction. By combining these two theoretical models, this study offers a more comprehensive framework that deepens our understanding of social media usage behavior and serves as a powerful theoretical tool for studying complex user behaviors. This integration bridges the gap between user motivations and post-adoption behaviors, advancing theoretical development in social media and tourism behavior research.

***Third, theoretical insights into data analysis methods*:** Another significant theoretical contribution of this study is its innovative data analysis methods. By integrating SEM and ANN, this study addresses the limitations of relying solely on a single analytical model to capture complex relationships. SEM effectively analyzes linear relationships between variables, tests hypotheses, and identifies causal relationships. However, it has limitations in capturing nonlinear interactions. ANN complements SEM by identifying nonlinear and non-compensatory relationships, enhancing predictive accuracy. Integrating these techniques advances methodological approaches in data analysis and provides a powerful tool for future studies investigating complex behavioral patterns. This methodological innovation contributes to theoretical discussions by demonstrating how advanced analytical techniques can uncover the nuanced aspects of user behavior.

***Fourth, expanding the theoretical scope of tourism research*:** While most existing tourism studies focus on traditional media or destination attractiveness, this study provides a new theoretical perspective by examining the influence of social media on RI in the sports tourism. By analyzing factors such as information quality, perceived enjoyment, and eWOM on social media, this study reveals how digital platforms enhance user experience, increase satisfaction, and stimulate RI. These findings challenge the conventional focus on the physical and tangible aspects of tourism destinations, highlighting the growing importance of digital platforms in shaping tourism behavior. This study contributes to the theoretical foundation for applying social media in tourism marketing. It lays the groundwork for future research on the role of other digital tools in shaping tourism behavior.

#### 5.1.2. Practical implications.

This study provides concrete strategic guidance and empirical support for tourism destination managers, operators, and policymakers by revealing the crucial role of social media in enhancing university students’ RI in sports tourism.

First, leveraging eWOM to enhance RI. The findings indicate that eWOM significantly impacts university students’ RI in sports tourism. Therefore, tourism destination managers should actively guide user-generated content on social media platforms, mainly through word-of-mouth marketing strategies that encourage positive feedback and recommendations. For instance, they can implement incentive mechanisms such as lotteries, discount coupons, or exclusive offers to motivate tourists to share their travel experiences and photos on social media. Additionally, official social media accounts should regularly feature positive visitor reviews and recommendations to strengthen potential visitors’ trust. Moreover, promptly responding to and addressing negative reviews can demonstrate a destination’s commitment to service improvement, ultimately enhancing its overall reputation and increasing visitor loyalty.

Second, information quality should be optimized to enhance tourist decision-making support. The findings indicate that information quality significantly influences tourists’ decision-making and RI. Therefore, tourism destinations should provide high-quality, authentic, and up-to-date information to help potential visitors better plan their travel experiences. For example, destinations should utilize social media platforms to share detailed and accurate information on facilities, activity schedules, and transportation guides to ensure practicality and reliability. Additionally, incorporating visual content such as images, videos, and virtual tours can effectively showcase the unique features and attractions of the destination, enhancing both the appeal and credibility of the information.

Third, enhancing perceived entertainment to improve tourist satisfaction. The study indicates that the perceived entertainment value of social media indirectly enhances RI by increasing tourist satisfaction. Therefore, tourism operators should design more engaging and interactive content to attract young tourists and improve their travel experiences. Specific strategies include: First, hosting interactive activities on social media, such as quizzes, polls, and challenges, to increase user engagement and entertainment. Second, short-video platforms (e.g., TikTok, Instagram) and live-streaming features can showcase real-time events and unique destination experiences, capturing tourists’ attention and interest.

Fourth, providing data support for policymakers. This study offers valuable data insights for government agencies and policymakers by highlighting the critical role of social media in enhancing RI in sports tourism. Based on the findings, policymakers can implement the following measures: First, tourism destinations can be encouraged to leverage social media for promotion by providing relevant training and technical support. Second, policy incentives (e.g., tax benefits subsidies) should be implemented to motivate tourists to share positive reviews and recommendations on social media. Finally, social media feedback can be utilized to optimize destination services and infrastructure, thereby improving management efficiency and promoting the sustainable development of the local economy.

### 5.2. Limitations and future work

First, the data collection for this study was limited to three universities in Hubei Province, China. This geographical limitation may restrict the generalizability of the findings, as university students in Hubei may have certain unique cultural and social characteristics that do not fully represent students across the country. Future research can expand the data collection to include students from a broader range of regions, especially from different provinces and areas, to verify the general applicability of the study’s results. By increasing the geographical diversity of the sample, the findings will become more comprehensive and possess stronger external validity.

Second, this study employed a cross-sectional research design, collecting data from participants at a single point in time. While this design can reveal correlations between variables, it cannot explore causal relationships or dynamic processes over time. As a result, the study cannot demonstrate the long-term effects of social media use on university students’ RI in sports tourism. Future research could adopt a longitudinal research design, collecting data over multiple time points to examine the temporal effects of social media use on RI. This approach would provide a more comprehensive understanding of the dynamic changes and long-term impacts.

Third, the variance explained by the model for university students’ RI in sports tourism is 71.7%. While this represents a substantial level in social science research, it still suggests that about 30% of the variance remains unexplained. Future research could consider introducing additional relevant variables, such as social support, personality traits, or cultural background, which might further enhance the model’s explanatory power. Moreover, employing more advanced statistical techniques, such as combining SEM with other non-linear analytical tools, could improve understanding social media’s impact and enhance the model’s predictive accuracy.

## 6. Conclusion

The purpose of this study was to comprehensively understand the influence of social media on university students’ RI in sports tourism. To achieve this goal, the research model integrated the ECM and the U&GT to explain how social media affects RI. Empirical data were collected from 435 university students from three Hubei Province. The results of SEM and ANN analyses showed that eWOM, information quality, and perceived enjoyment are key predictors of university students’ RI in sports tourism. However, the effect of perceived usefulness on RI is not significant. These predictor variables explain 71.7% of the variance in university students’ RI to a destination. Furthermore, the results of the ANN analysis revealed that eWOM is the strongest predictor, followed by information quality, satisfaction, perceived enjoyment, and perceived usefulness. This study provides a strong reference for future research on university students’ sports tourism behavior and offers practical recommendations for tourism marketers and destination managers. This study has some limitations regarding the sample source, cross-sectional design, and the need for additional constructs to improve explanatory power.

## Supporting information

S1 Table Supplementary results including descriptive statistics, measurement validation, and structural model analysis (Tables 1–9). (DOCX)

S1 File Appendix A document. (DOC)

S2 File Data. (SAV)
