# Supplementary material for: The impact of social media on university students’ revisit intention in sports tourism: A hybrid method based on SEM and ANN
Source: PLoS One. 2025 Apr 29;20(4):e0321999. doi: 10.1371/journal.pone.0321999 (PMC12040268; doi:10.1371/journal.pone.0321999)
Supplement: S1 Table — (DOCX) [file pone.0321999.s001.docx]

Table 1. Demographic Characteristics of the Sample

| Demographic | Category | Number | Percentage |
| --- | --- | --- | --- |
| Gender | Male | 187 | 42.99% |
|  | Female | 248 | 57.01% |
| Grade | Freshmen | 154 | 35.40% |
|  | Sophomore | 159 | 36.55% |
|  | Junior | 74 | 17.01% |
|  | Senior | 48 | 11.03% |
| Major | Sports Major | 130 | 29.89% |
|  | Non-Sports Major | 305 | 70.11% |

Table 2. Reliability and AVE

| Constructs | Items | Outer loadings | Cronbach's α | CR | AVE |
| --- | --- | --- | --- | --- | --- |
| IQ | IQ1 | 0.866 | 0.863 | 0.954 | 0.777 |
|  | IQ2 | 0.896 |  |  |  |
|  | IQ3 | 0.895 |  |  |  |
| PE | PE1 | 0.879 | 0.900 | 0.916 | 0.785 |
|  | PE2 | 0.898 |  |  |  |
|  | PE3 | 0.908 |  |  |  |
|  | PE4 | 0.823 |  |  |  |
| PU | PU1 | 0.79 | 0.864 | 0.931 | 0.770 |
|  | PU2 | 0.821 |  |  |  |
|  | PU3 | 0.868 |  |  |  |
|  | PU4 | 0.888 |  |  |  |
| RI | RI1 | 0.861 | 0.875 | 0.907 | 0.71 |
|  | RI2 | 0.914 |  |  |  |
|  | RI3 | 0.907 |  |  |  |
| SAT | SAT1 | 0.862 | 0.898 | 0.923 | 0.800 |
|  | SAT2 | 0.872 |  |  |  |
|  | SAT3 | 0.875 |  |  |  |
|  | SAT4 | 0.893 |  |  |  |
| eWoM | eWoM1 | 0.898 | 0.942 | 0.929 | 0.766 |
|  | eWoM2 | 0.904 |  |  |  |
|  | eWoM3 | 0.901 |  |  |  |
|  | eWoM4 | 0.885 |  |  |  |
|  | eWoM5 | 0.849 |  |  |  |
|  | eWoM6 | 0.85 |  |  |  |

Table 3. Discriminant Validity (Fornell-Larcker Criteria)

|  | eWoM | IQ | PE | PU | RI | SAT |
| --- | --- | --- | --- | --- | --- | --- |
| eWoM | **0.881** |  |  |  |  |  |
| IQ | 0.679 | **0.886** |  |  |  |  |
| PE | 0.738 | 0.713 | **0.878** |  |  |  |
| PU | 0.714 | 0.657 | 0.779 | **0.843** |  |  |
| RI | 0.804 | 0.716 | 0.723 | 0.698 | **0.895** |  |
| SAT | 0.838 | 0.779 | 0.745 | 0.74 | 0.779 | **0.875** |

*Note: The bolded values on the diagonal represent the square roots of the AVE for each construct.*

Table 4. Discriminant Validity (HTMT Criteria)

|  | eWoM | IQ | PE | PU | RI | SAT |
| --- | --- | --- | --- | --- | --- | --- |
| eWoM |  |  |  |  |  |  |
| IQ | 0.751 |  |  |  |  |  |
| PE | 0.802 | 0.809 |  |  |  |  |
| PU | 0.788 | 0.755 | 0.881 |  |  |  |
| RI | 0.883 | 0.823 | 0.814 | 0.799 |  |  |
| SAT | 0.816 | 0.883 | 0.828 | 0.834 | 0.877 |  |

Table 5. VIF

|  | eWoM | IQ | PE | PU | RI | SAT |
| --- | --- | --- | --- | --- | --- | --- |
| eWoM |  |  |  |  | 3.888 | 2.656 |
| IQ |  |  |  |  | 2.84 | 2.318 |
| PE |  |  |  |  | 3.413 | 3.407 |
| PU |  |  |  |  | 3.021 | 2.909 |
| RI |  |  |  |  |  |  |
| SAT |  |  |  |  | 4.075 |  |

Table 6. Path Analysis and Hypothesis Testing

| **Hypothesis** | **β coefficients** | **Standard deviation** | **T Statistics** | **P Values** | **Results** |
| --- | --- | --- | --- | --- | --- |
| IQ → RI | 0.198 | 0.047 | 4.23 | 0.000 | Supported |
| IQ → SAT | 0.321 | 0.039 | 8.199 | 0.000 | Supported |
| PE → RI | 0.11 | 0.054 | 2.042 | 0.041 | Supported |
| PE → SAT | 0.037 | 0.069 | 0.536 | 0.592 | Not Supported |
| PU → RI | 0.094 | 0.055 | 1.696 | 0.090 | Not Supported |
| PU → SAT | 0.148 | 0.05 | 2.963 | 0.003 | Supported |
| SAT → RI | 0.116 | 0.066 | 4.247 | 0.000 | Supported |
| eWoM → RI | 0.423 | 0.058 | 7.299 | 0.000 | Supported |
| eWoM → SAT | 0.493 | 0.051 | 9.597 | 0.000 | Supported |

Table 7. R^2^ and Q^2^

| Constructs | R^2^ | Q^2^ |
| --- | --- | --- |
| SAT | 80.3% | 0.607 |
| RI | 71.7% | 0.565 |

Table 8. Root mean square of error values.

| Training | | | Testing | | | Total samples |
| --- | --- | --- | --- | --- | --- | --- |
| N | SSE | RMSE | N | SSE | RMSE |  |
| 296 | 2.8409 | 0.0980 | 139 | 1.2085 | 0.0932 | 435 |
| 315 | 2.6609 | 0.0919 | 120 | 1.6093 | 0.1158 | 435 |
| 294 | 2.7396 | 0.0965 | 141 | 1.3248 | 0.0969 | 435 |
| 292 | 2.8469 | 0.0987 | 143 | 1.367 | 0.0978 | 435 |
| 298 | 2.8923 | 0.0985 | 137 | 1.201 | 0.0936 | 435 |
| 314 | 3.0965 | 0.0993 | 121 | 1.0382 | 0.0926 | 435 |
| 297 | 2.6916 | 0.0952 | 138 | 1.4360 | 0.1020 | 435 |
| 304 | 2.4602 | 0.0900 | 131 | 1.540 | 0.1084 | 435 |
| 307 | 2.8962 | 0.0971 | 128 | 1.251 | 0.0989 | 435 |
| 317 | 3.0961 | 0.0988 | 118 | 1.322 | 0.1058 | 435 |
| Mean | 2.822116667 | 0.0964 | Mean | 1.3297 | 0.1005 |  |
| Sd |  | 0.0032 | Sd |  | 0.0075 |  |

Table 9. Sensitivity analysis

| ANN | eWoM | IQ | PE | PU | SAT |
| --- | --- | --- | --- | --- | --- |
| ANN 1 | 1.000 | 0.577 | 0.319 | 0.343 | 0.357 |
| ANN 2 | 1.000 | 0.465 | 0.227 | 0.282 | 0.261 |
| ANN 3 | 1.000 | 0.405 | 0.384 | 0.106 | 0.355 |
| ANN 4 | 1.000 | 0.575 | 0.344 | 0.192 | 0.186 |
| ANN 5 | 1.000 | 0.582 | 0.564 | 0.397 | 0.594 |
| ANN 6 | 1.000 | 0.282 | 0.256 | 0.308 | 0.389 |
| ANN 7 | 1.000 | 0.402 | 0.306 | 0.285 | 0.294 |
| ANN 8 | 0.898 | 0.448 | 0.688 | 0.418 | 1.000 |
| ANN 9 | 1.000 | 0.394 | 0.412 | 0.197 | 0.529 |
| ANN 10 | 1.000 | 0.534 | 0.420 | 0.569 | 0.243 |
| Mean importance | 0.990 | 0.466 | 0.392 | 0.310 | 0.421 |
| Normalized importance (%) | 100.0% | 47.1% | 39.6% | 31.3% | 42.5% |
