# Supplementary material for: The impact of social media on university students’ revisit intention in sports tourism: A hybrid method based on SEM and ANN
Source: PLoS One. 2025 Apr 29;20(4):e0321999. doi: 10.1371/journal.pone.0321999 (PMC12040268; doi:10.1371/journal.pone.0321999)
Supplement: S1 File — (DOC) [file pone.0321999.s002.doc]

| Constructs | Items | Source |
| --- | --- | --- |
| Perceived usefulness | 1.I believe that social media enables me to complete sports tourism plans more quickly. | (Al-Adwan et al.,  2023) |
|  | 2.I believe that social media improves the efficiency of accessing sports tourism information. |  |
|  | 3.I believe that social media enhances the quality of sports tourism. |  |
|  | 4.I believe that social media is very useful in sports tourism. |  |
| Perceived enjoyment | 1.Using social media makes sports tourism more enjoyable. | (Al-Adwan et al.,  2023) |
|  | 2.Using social media makes sports tourism more of a pleasure. |  |
|  | 3.Using social media makes sports tourism more entertaining. |  |
|  | 4.Social media makes my leisure time (such as weekends or holidays) more fun. |  |
| Information quality | 1.I believe that the information provided by social media is reliable and accurate. | (Idkhan & Idris, 2023) |
|  | 2.I believe that social media presents information clearly and understandably. |  |
|  | 3.I believe that the information on social media is novel and comprehensive. |  |
| Satisfaction | 1.I am satisfied with the overall functionality of social media. | (Idkhan & Idris, 2023) |
|  | 2.I am satisfied with sports tourism after using social media. |  |
|  | 3.I believe that using social media can enhance my knowledge and skills in sports tourism. |  |
|  | 4.I believe that social media offers more options for sports tourism. |  |
| Electronic word of mouth | 1.Through social media, electronic word-of-mouth (e.g., reviews of a sports tourism destination on Douyin videos) can increase university students' willingness for sports tourism. | (Xu et al., 2024) |
|  | 2.Through social media, electronic word-of-mouth can encourage university students' participation in sports tourism activities. |  |
|  | 3.The electronic word-of-mouth on social media (e.g., Douyin reviews) can enhance university students' willingness for sports tourism. |  |
|  | 4.I believe that electronic word-of-mouth helps in choosing to use social media for sports tourism. |  |
|  | 5.I have reviewed electronic word-of-mouth on social media and felt satisfied. |  |
|  | 6.I believe that online reviews and recommendations related to sports tourism on social media are helpful to me. |  |
| Revisit intention | 1.I believe that social media is my first choice for sports tourism. | (Nazarian et al., 2024) |
|  | 2.I will use social media again on my next sports tourism trip. |  |
|  | 3.I will continue to use social media for sports tourism in the foreseeable future. |  |
